# Does the c.-14C>T Mutation in the *IFITM5* Gene Provide Identical Phenotypes for Osteogenesis Imperfecta Type V? Data from Russia and a Literature Review

**DOI:** 10.3390/biomedicines10102363

**Published:** 2022-09-22

**Authors:** Anton Tyurin, Elena Merkuryeva, Aliya Zaripova, Tatyana Markova, Tatyana Nagornova, Ilya Dantsev, Dina Nadyrshina, Ekaterina Zakharova, Rita Khusainova

**Affiliations:** 1 Internal Medicine Department, Bashkir State Medical University, 450008 Ufa, Russia; 2 Research Centre for Medical Genetics, 115522 Moscow, Russia; 3 Laboratory of Human Molecular Genetics, Institute of Biochemistry and Genetics, 450000 Ufa, Russia; 4 Veltischev Research and Clinical Institute for Pediatrics, Pirogov Russian National Research Medical University, 125412 Moscow, Russia; 5 Healthy Longevity Center, Bashkir State University, 450008 Ufa, Russia; 6 Medical Genetics Department, Bashkir State Medical University, 450008 Ufa, Russia

**Keywords:** osteogenesis imperfecta (incomplete), interferon-induced transmembrane protein 5 gene, mutations, polymorphism

## Abstract

Osteogenesis imperfecta (OI) is a large group of genetically heterogeneous diseases resulting from decreased bone density and an abnormal microarchitecture, which are clinically manifested by abnormal bone fractures. A distinctive clinical feature of this group of diseases is the presence of spontaneous fractures and skeletal deformities. However, the clinical manifestations of different types of OI are characterized by marked polymorphism with variable severity of skeletal and extra-skeletal features. Previous studies have shown that a mutation (c.-14C>T) in the *IFITM5* gene is responsible for autosomal dominant OI type V. However, the mutation has a variable expression pattern and marked clinical heterogeneity. In this study, a clinical and genetic analysis of 12 cases with molecularly confirmed OI type V from 12 unrelated families was performed. Significant clinical heterogeneity of the disease with the same molecular defect was detected. In six subjects (50%), there were no classic signs of OI type V (formation of a hyperplastic bone callus, calcification of the interosseous membrane and dislocation of the radial head). In all cases, the mutation occurred de novo.

## 1. Introduction

Osteogenesis imperfecta (ICD-10 code Q78.0; incomplete osteogenesis, “congenital bone failure and fragility” or Lobstein–Wrolick disease is the common name in the national literature) is a congenital metabolic bone disease. It is a clinically and genetically heterogeneous hereditary connective tissue disorder characterized by an abnormal bone structure, resulting in frequent fractures with characteristic disabling bone deformities, impaired growth and posture, and a wide range of associated respiratory, cardiovascular, neuromuscular, auditory and visual problems [1]. Clinical features can range from mild symptoms with few fractures to severe bone deformities and neonatal mortality [2]. The prevalence of OI in the USA and Europe ranges from 1:20,000 to 1:50,000 in the population [3,4]. OI is clinically categorized using the Sillence scale, which originally delineated four types of OI based on their clinical presentation. These “classical” OI types are associated with autosomal mutations in the genes encoding type I collagen (*COL1A1, COL1A2*) and still account for 85% to 90% of OI cases [5]. These mutations lead to structural or qualitative defects in the collagen protein, with reductions in collagen quantity (haploinsufficiency) typically producing milder forms of OI compared with structural mutants [6]. Based on the clinical phenotypes and later on the confirmation of molecular markers, types V and VI of the disease were distinguished.

Several groups of drugs are now being used to treat OI. Bisphosphonates, which act by inhibiting osteoclast activity and bone resorption, are the mainstay of pharmacologic treatment in pediatric patients with OI. Bisphosphonates have been shown to consistently improve bone mineral density in patients with OI [7] and, to some extent, reduce fracture incidence [8]. Denosumab, an anti-RANKL (receptor activator of nuclear factor kappa-B ligand) antibody that inhibits osteoclast differentiation and function, is considered to be a possible treatment for OI in a number of recently completed clinical trials (NCT03638128 and NCT02352753). Teriparatide, a PTH analogue (recombinant human parathyroid hormone) that induces anabolism in bone, significantly increased bone mineral density in adults with OI type I. Its clinical use is limited to adults and is restricted to 24 months duration. Sclerostin inhibitory antibody is an anabolic agent designed to target sclerostin, an inhibitor of bone formation via the canonical WNT signaling pathway, and is in a phase 2 clinical trial. Adult OI patients exhibited increased bone formation, decreased bone resorption and increased bone mineral density after a short-term dose-escalation trial with BPS804 anti-sclerostin antibody. Transforming growth factor beta (TGFβ) inhibition targets the excessive activation of TGFβ signaling that is implicated in regulating bone mass and fragility in OI [9,10]. Preclinical studies showed increased bone mass and bone strength in mouse models of OI treated with TGFβ inhibitory antibody [11]. The safety and efficacy of fresolimumab, a TGFβ inhibitory antibody, has been investigated in adult OI patients (NCT03064074).

One promising area is gene and cell therapy. Progenitor cell therapy via the transplantation of healthy progenitor stem cells has been proposed to specifically address the inherent bone fragility in OI. Gene therapy is also a promising therapeutic option, which involves the suppression of unfavorable or harmful transcripts, increased expression of healthy alleles, or repair of genetic abnormalities either transiently or permanently. For both types there will be an increasing need to establish causative mutations [12]. 

To date, 22 genetic variants of OI, corresponding to disease types I to XXII, have been described, and 20 genes responsible for their occurrence have been identified (OMIM^®^-An Online Catalog of Human Genes and Genetic Disorders. Available online: https://www.omim.org/ (accessed on 18 September 2022). The disease is inherited in both autosomal dominant and autosomal recessive types, with a predominance of the autosomal dominant type of inheritance with familial mosaicism, but there are also sporadic cases due to de novo mutations, the frequency of which has yet to be determined, and X-linked forms have also been found [5].

Mutations in the *IFITM5* gene, also called *BRIL* (bone-restricted IFITM-like protein), result in OI type V. Previous studies showed that the proportion of patients with OI type V can range from 5 to 10% of all cases [13,14]. Classic OI type V is characterized by hyperplastic callus formation after fractures, calcification of the interosseous membrane of the forearm, dislocation of the radial head and a reticular lamellar pattern observed on histological bone examination, but none of these features is pathognomonic for this type of OI, resulting in its pronounced clinical heterogeneity. In 2012, a heterozygous mutation (c.-14C>T) in the 5’-untranslated region (UTR) of the *IFITM5* gene was identified as the main cause of OI type V, greatly simplifying the diagnosis, and in 2014, a c.119C>T mutation was identified, with minor differences in the phenotype. According to the current classification, OI type V is characterized by an autosomal dominant inheritance. However, the questions about the frequency of this type of disease and clinical criteria for the diagnosis or exclusion of OI type V are still relevant. 

This study aimed to chart clinical and genetic characteristics of OI type V with identified pathogenic changes in the *IFITM5* gene in patients from Russia. 

## 2. Materials and Methods

We used deoxyribonucleic acid (DNA) samples from 230 patients with OI who had been observed in the Medical Genetics Research Center (Moscow, Russia) since 2017 and 62 patients with OI who had been observed in the Republican Clinical Genetics Center and Clinic of Bashkir State Medical University (Ufa, Republic of Bashkortostan) since 2008. To clarify the diagnosis, the following methods were used: genealogical analysis, clinical examination, neurological examination according to the standard technique with an assessment of the psychoemotional sphere, radiography and targeted panel sequencing consisting of 166 genes responsible for the development of hereditary skeletal pathology (see Supplementary Materials Table S1). 

As a result of mutation screening in all patients with OI by targeted next-generation sequencing (NGS), the c.-14C>T mutation in the *IFITM5* gene was detected in nine patients, and the Sanger method detected it in three patients.

Isolation of genomic DNA was carried out from whole blood using DNAEasy (QiaGen, Hilden, Germany) according to the manufacturer’s standard protocol. The concentration of DNA and DNA libraries were measured on a Qubit 2.0 instrument using reagents (Qubit Broad Range, Qubit High Sensitivity assay kits, Thermo Fisher Scientific, Waltham, MA, USA) from the manufacturer according to the standard protocol. For sample preparation, a technique based on multiplex polymerase chain reaction of target DNA regions was used. Next-generation sequencing was carried out on an Ion Torrent S5 sequencer with an average coverage of at least 80×; the number of targeted areas had a coverage ≥90–94%. To annotate the identified variants, nomenclature presented at DNA Recommendations. Available online: http://varnomen.hgvs.org/recommendations/DNA version 2.15.11 (accessed on 18 September 2022) was used. Sequencing data were processed using a standard automated algorithm from Ion Torrent.

To assess the population frequencies of identified variants, samples of the «1000 Genome» projects, ESP6500, and the Genome Aggregation Database v2.1.1 (Available online: https://gnomad.broadinstitute.org/ (accessed on 18 September 2022)) were used. To assess the clinical significance of the identified variants, the Online Mendelian Inheritance in Man (OMIM) database and the HGMD^®^ Professional pathogenic variants database version 2021.3 were used. Assessment of the pathogenicity and causality of genetic variants was carried out in accordance with international recommendations for the interpretation of data obtained by massive parallel sequencing [15].

Validation of the identified variants in probands and genotyping of siblings and parents were carried out by automated Sanger sequencing according to the manufacturer’s protocol on an ABIPrism 3500xl device (Applied Biosystems, Waltham, MA, USA). The primer sequences were selected according to the reference sequence of the *IFITM5* gene target (NM_001025295.3), forward (5′ to 3′): ACCAGTCTGAGTGTGGAAGAGA, reverse: AGTGTGAGGGCTGTGTGGG.

## 3. Results

We sampled twelve patients with OI type V with an identified c.-14C>T mutation in the *IFITM5* gene. Nine patients with OI type V were identified by screening 230 patients with OI at the Medical Genetics Research Center (Moscow) (frequency 3.9%), and the remaining three patients came from the Republic of Bashkortostan (Ufa), where 62 patients with OI from 52 families were registered (frequency 5.7%). The difference in incidence can probably be ascribed to the fact that the Moscow center receives patients with suspected OI, while the Ufa center sees patients with an established diagnosis. The average incidence of OI type V in the entire patient sample was 4.1%.

Across all cases, the clinical symptomatology was highly heterogeneous, and the diagnosis was established based on molecular genetic studies. In patients 1, 10 and 11, type V was suspected on clinical examination, and a mutation in the *IFITM5* gene was identified by the Sanger method. In the remaining nine unrelated patients with OI, an alteration to the *IFITM5* gene was found by mutation screening using targeted NGS. Demographics, fracture information and the age at treatment initiation are summarized in Table 1. 

In all our patients, the mutation was observed de novo. No cases of autosomal dominant inheritance were identified, which contradicts the literature. Based on previous studies of OI heritability in general, the frequency of de novo mutations was 41.03% in South Korea [16], 56.16% in Estonia, Ukraine and Vietnam [17], and in a study of 135 patients from China it was 37.78%. In the most recent study, the non-penetrance rate of hereditary proband parents was as high as 25.64%. Thus, some sick hereditary proband parents had very mild or even asymptomatic symptoms, which can lead to missed diagnosis and increased rates of de novo false-positive mutations if parental gene testing is not performed [18]. Significant clinical variability was observed in the twelve unrelated patients with OI type V. All subjects had a low height (SD score: 1.1 to −5.4). The number of fractures ranged from 3 to 50, and the age at the time of the first fracture ranged from birth to 2.5 years. Compression fractures of the vertebrae were observed in five patients (41.7%). Eight patients were treated with bisphosphonates. Bone mineral density (BMD) has been shown to vary widely, but our data were difficult to interpret because of the use of bisphosphonates in some patients and different methods of BMD measuring. In this study, one patient showed no decrease in BMD, possibly due to the early initiation of bisphosphonate treatment. Eight (66.7%) patients could walk without assistance, while four (33.3%) patients were unable to walk independently and used a wheelchair.

The clinical signs are shown in Table 2. A few studies reported that blue sclerae could be seen in OI type V patients, e.g., Shapiro et al. [19], who reported 2 out of 17 patients with blue sclerae. In our case, five patients (41,7%) had bluish sclerae. Hearing problems and dentinogenesis imperfecta did not occur in any of the subjects. Patient 1 had thin enamel and dental caries, and patient 2 had dental anomalies in the form of crowding and irregular growth of the teeth, but with enamel of a normal color and no changes in the tooth shape noted. Patient 3 showed signs of enamel damage and frequent dental caries, and we noted crowding and irregular growth of the teeth. In addition to these findings, patient 1 had a level III general speech impairment and patient 4 had an activity and attention deficit syndrome. No other patients had issues with their cognition or speech.

**Figure 1:**
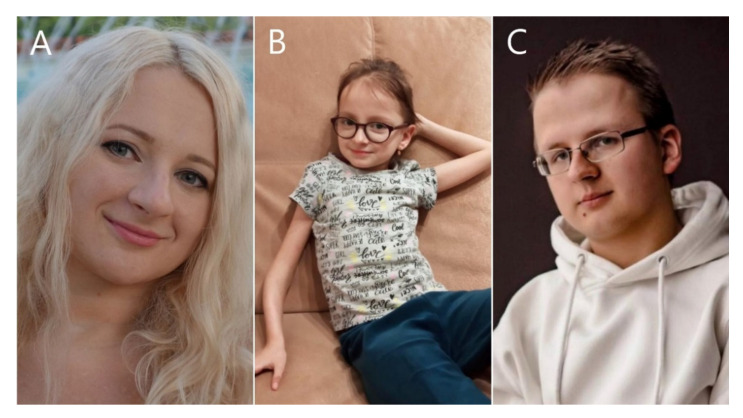
Photos of patients 8 (**A**), 10 (**B**) and 11 (**C**) with specific facial phenotypes—a thin nasal back with a pointed tip, a small pointed chin, thin lips, almond-shaped eyes, hypoplasia of the middle third of the face and large protruding auricles.

In 2019, new specific facial features of OI type V in Chinese (Han Chinese) patients were described: wide-set eyes, flat nose, wide jaw, small mouth with thin lips and a short broad forehead [20]. None of our patients had the described phenotype. We observed specific facial features—a thin nasal back with a pointed tip, a small pointed chin, thin lips, almond-shaped eyes, hypoplasia of the middle third of the face in four patients (4, 8, 10, 11), protruding frontal cusps in two patients (4, 10) and large protruding auricles. Examples of our patients’ facial phenotypes are shown in Figure 1.

In our study, nine subjects (75%) suffered from joint contractures: the elbow joints were affected in five patients, the elbow and knee joints in patients 8 and 10 and the ankle joints in patient 3. Patient 12 had flexion-adductor contractures of both hip joints. The occurrence of contractures was probably due to bone deformities after fractures. Patient 2 also had hyperpronation in the right elbow joint. Severe joint hypermobility was observed only in patient 2, and in patients 1, 7 and 8, only in the hand joints. The radiological features of patients with OI type V are summarized in Table 3. Severe calcification of the interosseous membranes was observed in only four patients (33.3%), and in patient 2 there was slight ossification of the forearm and tibia membranes. Hyperplastic callus after fractures occurred in four patients (33.3%). Eight other patients had no callus formation after fractures (66.7%). Nine patients (75%) had mild to severe scoliosis. All had curvature of the long tubular bones.

**Figure 2:**
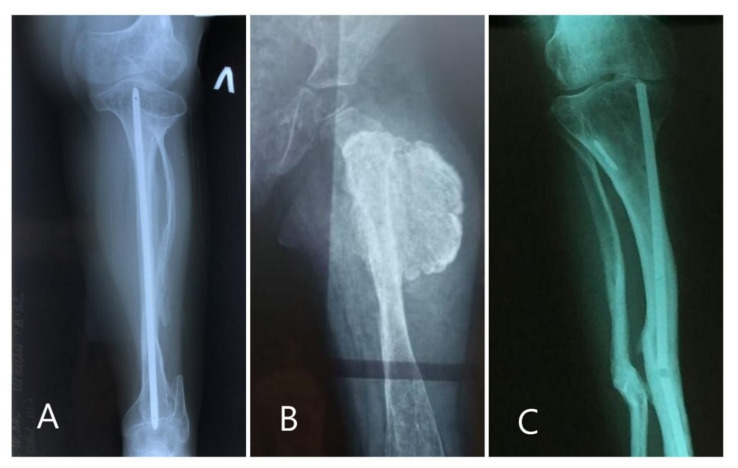
X-ray of patients 8 (**A**), 11 (**B**) and 12 (**C**). We see the absence of hypertrophic callus at the fracture site in patient 8, the presence of hypertrophic callus in patient 11 with the classic triad of signs and the absence of ossification of the interosseous membrane in patient 12.

In this study, we examined the clinical features of twelve individuals from twelve unrelated families diagnosed with OI type V and identified a pathogenetic c-14C>T mutation in the *IFITM5* gene. Five patients show a classic clinical picture of OI type V, and patient 2 had an atypical clinical picture, similar to the clinical manifestations of Ehlers–Danlos disease. This patient had a previously undescribed mutation c.1903 C>T: p.Arg635 * in a heterozygous state in the laminin B3 (*LAMB3*) gene, mutations that are found in bullous epidermolysis and amylogenesis imperfecta [21], resulting in a milder clinical manifestation of OI type V. This patient had no skin problems, and though some dental anomalies were present, the dental condition did not require medical intervention until in the last year, when caries of several localizations appeared. Many types of OI are characterized by dentinogenesis imperfecta, and a defect in the *LAMB3* gene may underlie the molecular pathogenesis of this condition. Co-mutations in the *IFITM5* and *LAMB3* genes have not been described in the literature. It was only after a molecular genetic diagnosis that it became clear that the patient had OI type V. In five subjects (50%), there were no classic signs of OI type V (formation of hyperplastic bone callus, calcification of the interosseous membrane and dislocation of the radial head). Examples of images with radiological manifestations are shown in Figure 2. One patient (not among the twelve sampled) was identified as having the c.306delG(p.G102fs) mutation at birth, but no clinical data were available, so he was not included in the study. 

## 4. Discussion

### 4.1. Molecular Basis for OI Type V

The first references to the phenotypic features of OI type V, in particular, hypertrophic callus, were mentioned in the literature as early as 1908 [22], then occurring periodically in subsequent publications. In a series of 60 type V patients, 10 (17%) developed at least one hyperplastic callus before the age of 20 years [23,24]. At that time, no molecular marker for this type of disease had yet been identified, nor had a distinct type itself been identified. One of the first reviews of OI type V was in an article by F.H. Glorieux and F. Rauch from 2004, where an attempt was made to systematize the information available at that time [25]. The familial occurrence of hyperplastic callus in OI with an autosomal dominant inheritance pattern has been described in some cases where additional features, such as a calcified interosseous membrane and irregular collagen fibril diameter, were associated with hyperplastic callus formation [26]. A few publications described individual clinical cases of patients with OI type V. Fleming et al. described a case of a patient with classical clinical manifestations and noted a positive effect of bisphosphonate therapy at an early age [27]. The first description of 12 patients with OI type V in Asian populations was in 2006 [28]. 

A breakthrough in the diagnosis of OI type V occurred in 2012 when a mutation in the *IFITM5* gene was first identified and described [29]. The mutation adds five residues to the N-terminus of *IFITM5*. This gene, also known as bone-restricted interferon-induced transmembrane protein-like protein or *BRIL*, encodes a protein with a function in bone formation and is located on chromosome 11p15.5. This protein is highly enriched in osteoblasts and is considered important during osteoblast formation in mice [30]. It encodes 132 amino acid proteins and has two transmembrane domains. The mouse *Ifimt5* gene has 88% similarity with the human *IFITM5* gene. The expression pattern of *Ifitm5* during embryonic development is similar to that observed in Osterix (Sp7), the human ortholog of which mutates into a rare autosomal recessive form of OI. Available expression data from adult rodents prove a role for *Ifitm5* not only in bone formation during embryogenesis but also during postnatal development. *Ifitm5−* mice have long bones, 15–25% shorter at birth than *Ifitm5+* mice, and they are sometimes severely curved, a symptom that partly passes by adulthood. Increased bone fragility or hyperplastic mosaic formation has not been reported for *Ifitm5−* deficient mice. Other bone mass deficiencies observed in newborn mice, such as a less calcified mandible and a thinner skull, disappeared after 5 weeks [31]. Later studies suggest a regulatory role for *BRIL* in the production of pigment epithelium, a multifunctional protein that plays a crucial role in bone mineralization and which is absent in the recessive clinical form, OI type VI [32]. The hyperplastic callus formation and calcification of the interosseous membrane observed in OI type V may be due to the removal of this suppression. Hyperplastic callus formation may be due to excess mineralization caused by the facilitation of *IFITM5* suppression over the main system involved in mineralization, and calcification of the interosseous membrane may be due to the removal of *IFITM5* suppression at sites that are not normally ossified [33].

A second mutation in this gene, c.119C>T, was discovered in 2014 by researchers from Spain. They reported a 5-year-old female patient with prenatally diagnosed OI due to limb shortening who was heterozygous for this mutation. She did not have the typical features of OI type V, although there was limited pronation/supination of the forearms in addition to the characteristic linear thickening on long tubular bone radiographs [34]. Farber et al. (2014) cited an observation of a Caucasian woman with severe progressive OI without the typical features of V type, heterozygous for the S40L missense mutation in the *IFITM5* gene. At 25 years of age, she was extremely stunted with a body length of 87 cm and body weight of 33.3 kg, and she suffered from relative macrocephaly with a head circumference of 53.5 cm. She had facial phenotypes (a round face with a high convex forehead and bluish sclerae, along with dentinogenesis imperfecta) and skeletal phenotypes (a barrel chest with funnel-shaped deformity, curvature of long tubular bones, scoliosis and hyperlordosis). She had no dislocation of the radial head, hypertrophic bone callus formation, ossification of the interosseous membrane or dense metaphyseal bands. A biopsy of the iliac bone at age 7 years revealed broad bands of unmineralized osteoid and a scaly lamellar plate pattern, which is characteristic of OI type VI [35]. Another mutation, a de novo heterozygous missense mutation c.119C>G in the *IFITM5* gene, accompanied by the formation of skeletal abnormalities, was described by Lim et al. in 2019. The patient had severe OI: bilateral lower limb deformities and short body length (−2.4 SD), multiple femur and tibia fractures on both sides, multiple rib fractures and a slightly bluish scleral tinge. However, she had no fractures at the age of 10 years and no signs of osteoporosis on BMD testing, but she had a progressive deformity of the lower extremities [36]. Finally, a third possible pathogenic variant in the *IFITM5* gene has recently been identified. Wu et al. identifies a de novo heterozygous pathogenic variant of *IFITM5* (NM_001025295.2:c.-9C>A) carried by a newborn with a fracture in her right clavicle. According to American College of Medical Genetics and Genomics (ACMG) guidelines with minor adjustments, the *IFITM5* c.-9C>A variant was classified as likely pathogenic. Similar to the recurrent c.-14C>T variant, this variant was located in the evolutionarily conserved allele of the 5’-UTR of *IFITM5* resulting in a mutated *IFITM5* protein with additional amino acids (MEP-IFITM5) [37]. These mutations have not been identified in patients from Russia.

Research has been carried out to better understand the molecular pathogenesis of the changes that mutations in the *IFITM5* gene carry. Although *BRIL* expression has been demonstrated in bone tissues from rodents [30,38,39], humans [40,41], tammar wallaby [42], and chicken [43], a clear in vivo role for the protein is still largely elusive. One hypothesis put forward is that the acidic C-terminus of *BRIL* represents a calcium binding moiety, although no formal experimental validation has been performed [44,45]. Studies by Lietman et al. [46] demonstrated that osteoblast-specific overexpression of *BRIL* in a transgenic mouse model, under regulation of the collagen type I promoter, resulted in an absence of phenotypic consequences on the skeleton. Additionally, mice generated with the entire locus of the five *Ifitm* genes deleted (*IfitmDel*) did not appear to possess any gross skeletal abnormalities, although this phenotype was not directly characterized [47]. *IfitmDel* mice are comparable to wild-type littermates with regard to their size and weight as well as behavior at birth and are fertile as adults, although this has more recently been questioned with respect to the mouse genetic background [48]. The only formally described phenotypes in these animals are an increased sensitivity to viral infections [49,50] as well as the development of a moderate metabolic syndrome in adulthood [51]. 

Lu et al. found that *IFITM5* was not only expressed in bone, but also expressed in bone marrow, skin, pancreas and smooth muscle, and this is inconsistent with previous studies that *IFITM5* was only expressed in bone. The reason for this difference may be that the specific expression of *IFITM5* in bone is only in certain mammals [38]. In the zebrafish model, *IFITM5* was expressed in brain, muscle and liver tissues with no expression in bone; in the chicken model, its expression was high in muscle and liver, low in brain and ovary, but with none in bone [42]. Therefore, the expression level of *IFITM5* in bone is different in various species, indicating that the *IFITM5* gene may have functions other than bone mineralization [52].

### 4.2. Clinical Phenotypes of Patients with OI Type V

The overall incidence of OI type V in our study was 4.1%. This is broadly consistent with the results obtained by other researchers. In 50 patients from India, OI type V was detected in 2 cases (4%) [53], among 29 Malaysian patients—1 case (3.4%) [54], among 53 patients in the Japanese population—1 case (1.8%) [55] and among 39 patients from Turkey—2 cases (5.13%) [56]. OI type V has some unique clinical features; however, not all patients develop them. For example, half of the patients in our study did not have any of the classic features of OI type V (formation of a hyperplastic bone callus, calcification of the interosseous membrane and dislocation of the radial head). We compared the phenotypic manifestations of OI type V in our study with the literature data (Table 4).

Eight publications describing the follow-up of patients with OI type V were reviewed, with a total of 96 patients from different countries and ethnic groups. Patients varied widely in age; both children with active disease and their family members were examined. A mutation (c.-14C>T) in the *IFITM* gene was identified in all patients. Most of the literature indicates that patients with OI type V do not have the typical features of OI, such as blue sclerae and dentinogenesis imperfecta. However, there are isolated reports of patients with these phenotypic features. In our study, blue sclerae were observed in 41.7% of our patients. The frequency of extracellular signs (dentinogenesis imperfecta, hearing loss) in our study was generally similar to that in previous studies. In contrast, calcification of the interosseous membranes was not the most significant sign (41.7%), which differs significantly from the previously reported results in which this sign was found in the majority of patients examined. Dislocation of the radial head is found in the literature with a frequency ranging from 36% to 100% of patients; in the patients examined by us, only patient 11 had a dislocation. Meanwhile, hyperplastic callus is described in the literature in 65–77% of cases, but in our study, the percentage was much lower—hyperplastic callus was found in only four patients—with the same small percentage as that indicated by Dong et al. (2006). Restriction of forearm rotation was found in eight patients (66.7%), which was less than in the main results presented previously. The frequencies of long tubular bone deformities (100%), scoliosis (75%) and vertebral compression fractures (41.7%) were comparable to the results of other studies. An interesting question is the effect of OI therapy with bisphosphonates on the HC formation. While bisphosphonate therapy is the standard of care for most forms of OI [60], there is limited information regarding the effects of the therapy on HC, which is an integral component of OI Type V. In a study by Cheung et al. (2007) [61], in 23 patients with type V OI, pamidronate therapy was not found to influence the course of HC formation. In another study of 11 patients with OI type V, the response to pamidronate treatment was found to be the same as in other types of OI [57]. Exacerbation of HC formation on treatment with bisphosphonates was observed in one study [62,63].

An analysis of our study results and the literature raises several questions: is it possible that the classic clinical and radiological features of OI type V develop later? We think this is unlikely, as there are reports of patients as young as 2 months of age who have the classic phenotype of OI type V. Our oldest patient without classical signs of OI type V was 43 years old. Calcinosis of the interosseous membrane has previously been identified in all patients with OI type V older than 4 years [64]. In our study, there was no evidence of interosseous membrane calcification in patients 3 (24 yrs), 4 (4 yrs 9 months), 5 (6 yrs), 7 (4 yrs 6 months) and 12 (43 yrs). Can the development of these signs be prevented by early initiation of bisphosphonate therapy? The cohorts described in the literature included patients who started treatment early and still developed hyperplastic callus or calcification of the interosseous membranes. In our patients (1, 10, 11) with classic signs of OI type V, treatment was started at 2 yrs 6 months, 4 yrs 6 months and 9 yrs, respectively. This leads us to a third question: is this mutation specific to OI type V, or is it possible that this case is another example of marked phenotypic variation with no genotype–phenotype correlation in OI? If true, this suggests other factors may modify the expression of this mutation, such as polymorphisms in other genes. Given the high phenotypic variability of OI type V and the fact that all patients have the same *IFITM5* mutation, this would present an ideal condition under which to search for modifier genes in the next-generation sequencing era.

## 5. Conclusions

We obtained new information on the clinical manifestations of OI type V in patients from Russia. The question remains open as to the molecular cause of the clinical heterogeneity of this type of disease. It is likely that there are modifying factors leading to marked clinical heterogeneity of the disease and that a broader panel for targeted NGS should be applied to identify other (additional) molecular defects. We propose screening all patients without pathogenic variants in the *COL1A1* and *COL1A2* genes for the presence of pathogenic variants in the *IFITM5* gene, even if they do not have the typical clinical symptoms of OI type V.

## Figures and Tables

**Table 1:** Demographic characteristics of patients with OI type V.

Patient	1	2	3	4	5	6	7	8	9	10	11	12
Sex, M/F	M	F	F	M	F	M	F	F	F	F	M	F
Age at admission, year	7	25	24	4.75	6	1.8	4	32	2.5	9	23	42
Age of clinical diagnosis, year	2.5	25	14	1.7	1.25	1.3	3	2	0.9	2	2	4
Age of molecular diagnosis, year	3	25	24	3	2.5	1.5	3.5	32	1.4	4.2	23	42
Classical OI type V	+	−	−	−	−	−	−	+	+	+	+	−
Family history	*−*	−	−	*−*	*−*	*−*	*−*	*−*	*−*	*−*	*−*	*−*
Ethnicity	*B*	*R*	*R*	*R*	*R*	*R*	*R*	*R*	*R*	Mixed (*R + M*)	*R*	Mixed (*Ch + U*)
Height, (SD Score)	−2.1	1.4	1.1	−1.21	−2.67	−1.54	−2.04	−5.4	−1.03	−1.78	−3.54	−2.48
Weight, (SD Score)	−1.96	−3.1	−3.3	−0.84	−1.88	−0.26	−2.92	−0.66	−2.23	−3.13	−0.74	0.09
Age of 1st fracture, year	2.5	at birth	at birth	at birth	7	8	1.5	at birth	4	2	1	1.10
Fractures total	10	>15	>50	8	11	3	6	35	4	10	40	12
Age of BFT start, year	2.5	−	−	−	2.5	1.3	3	−	1.5	4.2	9	34
Mobility	+	+	− (wheelchair)	+	+	+	+	− (wheelchair)	+	+	− (wheelchair)	− (wheelchair)

BFT, bisphosphonate treatment; R, Russian; B, Bashkir; M, Mari; Ch, Chuvash; U, Ukrainian; classical OI type V—formation of a hyperplastic bone callus, calcification of the interosseous membrane and dislocation of the radial head.

**Table 2:** Clinical characteristics of patients with OI type V.

Patient	1	2	3	4	5	6	7	8	9	10	11	12
Blue sclera	+	+	−	−	+	+	+	−	−	−	−	−
Hearing loss	−	−	−	−	−	−	−	−	−	−	−	−
Dentinogenesis imperfecta	−	−	−	−	−	−	−	−	−	−	−	−
Vertebral fracture	+	+	+	+	+	−	−	−	−	−	−	−
Limited rotation of forearm	+	+	+	+	+	−	−	+	−	+	+	−
Speech disorder	+	−	−	−	−	−	−	−	−	−	−	−
Intellectual disability	−	−	−	−	−	−	−	−	−	−	−	−

**Table 3:** Radiographic characteristics of patients with OI type V.

Patient	1	2	3	4	5	6	7	8	9	10	11	12
Hypertrophic callus	+	−	−	−	−	−	−	−	+	+	+	−
CIM	+	+	−	−	−	−	−	+	−	+	+	−
Scoliosis	+	+	+	+	+	−	−	+	−	+	+	+
BMD (g/cm^2^)	n/a	0.653	arms = 0.910 legs = 0.697 body = 1.15	n/a	LS = 0.870 TB = 0.850	n/a	LS = 0.231	LS = 0.719 FN = 0.634	n/a	LS = 0.254	LS = 0.344	n/a
BMD Z score	radius −6.3; tibia −6.6	−0.5	−0.5	n/a	0.5	n/a	TB = −1.3 LS = −3.9	LS = −3.1 FN = −2.4	n/a	LS = −5.6	LS = − 3.6	n/a

TB, total bode; LS, lumbar spine; FN, femur neck; CIM, calcification of interosseous membrane; n/a, not available.

**Table 4:** Comparative analysis of the clinical characteristics of the studied patients versus literature data.

	Zeitlin, 2006 [57]	Dong, 2006 [28]	Cho, 2012 [41]	Shapiro, 2013 [19]	Lazarus, 2014 [15]	Brizola, 2015 [58]	Liu, 2016 [59]	Cao, 2019 [20]	Our Results
N	11	12	16	17	9	7	11	13	12
Populations	Canada	Korean	Korean	USA	Australia	Brazil	China	Mixed	Russians
Age, years (Me, Q1; Q3)	8.7 (1.8; 15)	25 (10; 45)	20 (8; 46)	23 (7; 36)	11 (8; 25)	18 (10; 51)	10 (3.3; 16)	16.5 (10; 39)	6.5 (4; 24)
Height (Z score) (Me, Q1; Q3)	−2.6 (−6.0; 0.7)	0.12 (−1.4; 0.62)	−0.94 (−2.43; 0.2)	−2.9 (−4.3; −1.5)	−2.37 (−3; −1.1)	n/a	−2.2 (−3.7; −0.5)	n/a	−2.09 (−3;1; −0.8)
Blue sclera, n (%)	n/a	0	0	2 (11.7)	0	2 (28.6)	0	0	5 (41.7)
DI, n (%)	0	0	0	n/a	0	0	0	1 (7,7)	0
HC, n (%)	7 (64)	1 (8.3)	9 (56.2)	9 (53)	5 (55.5)	3 (42.8)	10 (91)	5 (38.4)	4 (33.3)
CIM, n (%)	11 (100)	12 (100)	16 (100)	13 (76.4)	9 (100)	7 (100)	9 (81.8)	12 (92.3)	5 (41.7)
DRH, n (%)	4 (36)	10 (83.3)	9 (56.2)	14 (82.3)	9 (100)	4 (57.1)	9 (81.8)	12 (92.3)	1 (8.3)
JC, n (%)	11 (100)	12 (100)	n/a	15 (88.2)	n/a	n/a	8 (72.7)	12 (92.3)	8 (66.7)
LBD, n (%)	10 (90)	n/a	n/a	n/a	n/a	n/a	n/a	6 (46.1)	12 (100)
VC, n (%)	11 (100)	n/a	n/a	n/a	n/a	7 (100)	6 (54.5)	7 (53.8)	5 (41.7)
Scoliosis, n (%)	8 (73)	4 (33.3)	10 (62.5)	13 (76.4)	n/a	6 (85.7)	4 (36.3)	7 (53.8)	9 (75)

DI, dentinogenesis imperfecta; HC, hypertrophic callus; CIM, calcification of interosseous membrane; DRH, dislocation of radial head; JC, joint contractures; LBD, long bone deformities; VC, vertebral compression; n/a, not available.

## Data Availability

The additional data are available in Supplementary Materials.

## Supplementary Materials

The following supporting information can be downloaded at: https://www.mdpi.com/article/10.3390/biomedicines10102363/s1, Table S1: List of genes included in the panel of targeted sequencing “Pathology of connective tissue”.
