# Supplementary material for: Does the c.-14C>T Mutation in the IFITM5 Gene Provide Identical Phenotypes for Osteogenesis Imperfecta Type V? Data from Russia and a Literature Review
Source: Biomedicines. 2022 Sep 22;10(10):2363. doi: 10.3390/biomedicines10102363 (PMC9598403; doi:10.3390/biomedicines10102363)
Supplement: Supplementary file 1 [file biomedicines-10-02363-s001.zip › biomedicines-1876629-supplementary.pdf]

Table S1. List of genes included in the panel of targeted sequencing "Pathology of connective tissue"

№	*Gene*	Gene Omim	Disease name	Diseaes Omim	Type of inheritance
1	*ADAMTSL2*	612277	Geleophysic dysplasia 1	231050	AR
2	*AGPS*	603051	Rhizomelic chondrodysplasia punctata	600121	AR
3	*ALPL*	171760	Hypophosphatasia	adult 146300	AR, AD
3	*ALPL*	171760	Hypophosphatasia	childhood 241510	AR
3	*ALPL*	171760	Hypophosphatasia	infantile 241500	AR
3	*ALPL*	171760	Odontohypophosphatasia	146300	AR, AD
4	*ANKH*	605145	Craniometaphyseal dysplasia	123000	AD
4	*ANKH*	605145	Chondrocalcinosis	118600	AD
5	*ARSB*	611542	Mucopolysaccharidosis type 6 (Maroteaux-Lamy)	253200	AR
6	*ARSE*	300180	Chondrodysplasia punctata, X-linked recessive	302950	XLR
7	*ATP6V1B1 (ATP6B1)*	192132	Renal tubular acidosis with deafness	267300	AR
8	*ATP6V0A4*	605239	Renal tubular acidosis, distal, autosomal recessive; RTADR	602722	AR
9	*BMP1*	112264	Osteogenesis imperfecta, type X2I	614856	AR
10	*CA2*	611492	Osteopetrosis, autosomal recessive 3; OPTB3	169600	AD
11	*CLCN5*	300008	Proteinuria, low molecular weight, with hypercalciuric nephrocalcinosis	308990	XLR
11	*CLCN5*	300008	Dent disease	300009	XLR
11	*CLCN5*	300008	Hypophosphatemic rickets, X-linked recessive	300554	XLR
11	*CLCN5*	300008	Nephrolithiasis, type I	310468	XLR
12	*COL10A1*	120110	Metaphyseal dysplasia, Schmid type (MCS)	156500	AD
13	*COL11A1*	120280	Fibrochondrogenesis 1	228520	AR
13	*COL11A1*	120280	Intervertebral disc disease	603932	Н/Д
13	*COL11A1*	120280	Marshall syndrome	154780	AD
13	*COL11A1*	120280	Stickler syndrome type 1	604841	AD
14	*COL11A2*	120290	Deafness, autosomal dominant 13	601868	AD
14	*COL11A2*	120290	Deafness, autosomal recessive 53	609706	AR
14	*COL11A2*	120290	Fibrochondrogenesis 2	614524	AR, AD
14	*COL11A2*	120290	Otospondylomegaepiphyseal dysplasia	215150	AR
14	*COL11A2*	120290	Weissenbacher-Zweymuller syndrome	277610	AD
14	*COL11A2*	120290	Stickler syndrome, type 2I	184840	AD
15	*COL1A1*	120150	Osteogenesis imperfecta, type I	166200	AD
15	*COL1A1*	120150	Osteogenesis imperfecta, type 2	166210	AD
15	*COL1A1*	120150	Osteogenesis imperfecta, type 2I	259420	AD
15	*COL1A1*	120150	Osteogenesis imperfecta, type IV	166220	AD
15	*COL1A1*	120150	{Bone mineral density variation QTL, osteoporosis}	166710	AD
15	*COL1A1*	120150	Caffey disease	114000	AD
15	*COL1A1*	120150	Ehlers-Danlos syndrome, classic	130000	AD
15	*COL1A1*	120150	Ehlers-Danlos syndrome, type V2A	130060	AD
16	*COL1A2*	120160	Osteogenesis imperfecta, type 2	166210	AD
16	*COL1A2*	120160	Osteogenesis imperfecta, type 2I	259420	AD
16	*COL1A2*	120160	Osteogenesis imperfecta, type IV	166220	AD
16	*COL1A2*	120160	{Osteoporosis, postmenopausal}	166710	AD
16	*COL1A2*	120160	Ehlers-Danlos syndrome, cardiac valvular form	225320	AR
16	*COL1A2*	120160	Ehlers-Danlos syndrome, type V2B	130060	AD
17	*COL2A1*	120140	Avascular necrosis of the femoral head	608805	AD
17	*COL2A1*	120140	Achondrogenesis, type 2 or hypochondrogenesis	200610	AD
17	*COL2A1*	120140	Kniest dysplasia	156550	AD
17	*COL2A1*	120140	Osteoarthritis with mild chondrodysplasia	604864	AD
17	*COL2A1*	120140	Otospondylomegaepiphyseal dysplasia	215150	AR
17	*COL2A1*	120140	Platyspondylic skeletal dysplasia, Torrance type	151210	AD
17	*COL2A1*	120140	Legg-Calve-Perthes disease	150600	AD
17	*COL2A1*	120140	Stickler syndrome, type I	108300	AD
17	*COL2A1*	120140	Stickler sydrome, type I, nonsyndromic ocular	609508	AD

17	*COL2A1*	120140	Spondyloperipheral dysplasia	271700	AD
17	*COL2A1*	120140	SMED Strudwick type	184250	AD
17	*COL2A1*	120140	Spondyloepiphyseal dysplasia, Stanescu type	616583	AD
17	*COL2A1*	120140	SED congenita	183900	AD
17	*COL2A1*	120140	Czech dysplasia	609162	AD
17	*COL2A1*	120140	Epiphyseal dysplasia, multiple, with myopia and deafness	132450	AD
18	*COL9A1*	120210	Stickler syndrome, type IV	614134	Н/Д
18	*COL9A1*	120210	Epiphyseal dysplasia, multiple, 6	614135	AD
19	*COL9A2*	120260	?Stickler syndrome, type V	614284	AR
19	*COL9A2*	120260	Epiphyseal dysplasia, multiple, 2	600204	AD
20	*COL9A3*	120270	Epiphyseal dysplasia, multiple, 3, with or without myopathy	600969	AD
21	*COMP*	600310	Pseudoachondroplasia	177170	AD
21	*COMP*	600310	Epiphyseal dysplasia, multiple, 1	132400	AD
22	*CREB3L1*	616215	Osteogenesis imperfecta, type XVI	616229	AR
23	*CRTAP*	605497	Osteogenesis imperfecta, type V2	610682	AR
24	*CTSA*	613111	Galactosialidosis	256540	AR
25	*CYP27B1*	609506	Vitamin D-dependent rickets, type I	264700	AR
26	*CYP2R1*	608713	Rickets due to defect in vitamin D 25-hydroxylation	600081	AR
27	*DDR2*	191311	Spondylometaepiphyseal dysplasia, short limb-hand type	271665	AR
28	*DMP1*	600980	Hypophosphatemic rickets, AR	241520	AR
29	*DYM*	607461	Dyggve-Melchior-Clausen disease	223800	AR
29	*DYM*	607461	Smith-McCort dysplasia	607326	AR
30	*DYNC2H1*	603297	Short-rib thoracic dysplasia 3 with or without polydactyly	613091	AR
31	*EBP*	300205	MEND syndrome	300960	XLR
31	*EBP*	300205	Chondrodysplasia punctata, X-linked dominant	302960	XLD
32	*EHHADH*	607037	Fanconi renotubular syndrome 3	615605	AD
33	*ENPP1*	173335	Arterial calcification, generalized, of infancy, 1	208000	AR
33	*ENPP1*	173335	Cole disease	615522	AD
33	*ENPP1*	173335	{Diabetes mellitus, non-insulin-dependent, susceptibility to}	125853	AD
33	*ENPP1*	173335	{Obesity, susceptibility to}	601665	Mu (Multifactorial), AD, AR
33	*ENPP1*	173335	Hypophosphatemic rickets, autosomal recessive, 2	613312	Н/Д
34	*EVC*	604831	Weyers acrodental dysostosis	193530	AD
34	*EVC*	604831	Ellis-van Creveld syndrome	225500	AR
35	*EVC2*	607261	Weyers acrofacial dysostosis	193530	AD
35	*EVC2*	607261	Ellis-van Creveld syndrome	225500	AR
36	*FAH*	613871	Tyrosinemia, type I; TYRSN1	276700	AR
37	*FBN1*	134797	Acromicric dysplasia	102370	AD
37	*FBN1*	134797	Geleophysic dysplasia 2	614185	AD
37	*FBN1*	134797	Marfan lipodystrophy syndrome	616914	AD
37	*FBN1*	134797	MASS syndrome	604308	Н/Д
37	*FBN1*	134797	Aortic aneurysm, ascending, and dissection	Н/Д	Н/Д
37	*FBN1*	134797	Ectopia lentis, familial	129600	AD
37	*FBN1*	134797	Stiff skin syndrome	184900	AD
37	*FBN1*	134797	Weill-Marchesani syndrome 2, dominant	608328	AD
37	*FBN1*	134797	Marfan syndrome	154700	AD
38	*FGF23*	605380	Tumoral calcinosis, hyperphosphatemic, familial	211900	AR
38	*FGF23*	605380	Osteomalacia, tumor-induced	-	-
38	*FGF23*	605380	Hypophosphatemic rickets, autosomal dominant	193100	AD
39	*FGFR3*	134934	CATSHL syndrome	610474	AR, AD
39	*FGFR3*	134934	Achondroplasia	100800	AD
39	*FGFR3*	134934	Hypochondroplasia	146000	AD
39	*FGFR3*	134934	Colorectal cancer, somatic	114500	Н/Д
39	*FGFR3*	134934	Cervical cancer, somatic	603956	Н/Д
39	*FGFR3*	134934	LADD syndrome	149730	AD
39	*FGFR3*	134934	Muenke syndrome	602849	AD

39	*FGFR3*	134934	Nevus, epidermal, somatic	162900	Н/Д
39	*FGFR3*	134934	SADDAN	616482	AD
39	*FGFR3*	134934	Spermatocytic seminoma, somatic	273300	-
39	*FGFR3*	134934	Bladder cancer, somatic	109800	-
39	*FGFR3*	134934	Crouzon syndrome with acanthosis nigricans	612247	AD
39	*FGFR3*	134934	Thanatophoric dysplasia, type I	187600	AD
39	*FGFR3*	134934	Thanatophoric dysplasia, type 2	187601	AD
40	*FKBP10*	607063	Osteogenesis imperfecta, type XI	610968	AR
40	*FKBP10*	607063	Bruck syndrome 1	259450	AR
41	*FLNA*	300017	Heterotopia, periventricular	300049	XLD
41	*FLNA*	300017	Otopalatodigital syndrome, type I	311300	XLD
41	*FLNA*	300017	Otopalatodigital syndrome, type 2	304120	XLD
41	*FLNA*	300017	Intestinal pseudoobstruction, neuronal	300048	XLR
41	*FLNA*	300017	Cardiac valvular dysplasia, X-linked	314400	XLR
41	*FLNA*	300017	Congenital short bowel syndrome	300048	XLR
41	*FLNA*	300017	Melnick-Needles syndrome	309350	XLD
41	*FLNA*	300017	FG syndrome 2	300321	-
41	*FLNA*	300017	Terminal osseous dysplasia	300244	-
41	*FLNA*	300017	Frontometaphyseal dysplasia	305620	XLR
42	*FLNB*	603381	Atelosteogenesis, type I	108720	AD
42	*FLNB*	603381	Atelosteogenesis, type 2I	108721	AD
42	*FLNB*	603381	Boomerang dysplasia	112310	AD
42	*FLNB*	603381	Larsen syndrome	150250	AD
42	*FLNB*	603381	Spondylocarpotarsal synostosis syndrome	272460	AR
43	*FUCA1*	612280	Fucosidosis	230000	AR
44	*GALNS*	612222	Mucopolysaccharidosis type 4A (Morquio A)	253000	AR
45	*GLB1*	611458	Mucopolysaccharidosis type IVB (Morquio)	253010	AR
45	*GLB1*	611458	GM1-gangliosidosis, type I	230500	AR
45	*GLB1*	611458	GM1-gangliosidosis, type 2	230600	AR
45	*GLB1*	611458	GM1-gangliosidosis, type 2I	230650	AR
46	*GNPAT*	602744	Rhizomelic chondrodysplasia punctata	222765	AR
47	*GNPTAB*	607840	Mucolipidosis 2 alpha/beta	252500	AR
47	*GNPTAB*	607840	Mucolipidosis 2I alpha/beta	252600	AR
48	*GNPTG*	607838	Mucolipidosis 2I gamma	252605	AR
49	*GNS*	607664	Mucopolysaccharidosis type 3D (Sanfilippo D)	252940	AR
50	*GPX4*	138322	Spondylometaphyseal dysplasia, Sedaghatian type	250220	AR
51	*GUSB*	611499	Mucopolysaccharidosis type 7 (Sly)	253220	AR
52	*HGSNAT*	610453	Mucopolysaccharidosis type 2IC (Sanfilippo C)	252930	AR
52	*HGSNAT*	610453	Retinitis pigmentosa 73	616544	AR
53	*HNF4A*	600281	Fanconi renotubular syndrome 4, with maturity-onset diabetes of the young	616026	AD
53	*HNF4A*	600281	MODY, type I	125850	AD
53	*HNF4A*	600281	{Diabetes mellitus, noninsulin-dependent}	125853	AD
54	*HSPG2*	142461	Dyssegmental dysplasia, Silverman-Handmaker type	224410	AR
54	*HSPG2*	142461	Schwartz-Jampel syndrome, type 1	255800	AR
55	*IDS*	300823	Mucopolysaccharidosis type 2 (Hunter)	309900	XLR
56	*IDUA*	252800	Mucopolysaccharidosis Ih	607014	AR
56	*IDUA*	252800	Mucopolysaccharidosis Ih/s	607015	AR
56	*IDUA*	252800	Mucopolysaccharidosis Is	607016	AR
57	*IFITM5*	614757	Osteogenesis imperfecta, type V	610967	AD
58	*IFT122*	606045	Cranioectodermal dysplasia 1	218330	AR
59	*IFT140*	614620	Short-rib thoracic dysplasia 9 with or without polydactyly	266920	AR
60	*IFT172*	607386	Short-rib thoracic dysplasia 10 with or without polydactyly	615630	AR
60	*IFT172*	607386	Retinitis pigmentosa 71	616394	AR
61	*IFT43*	614068	Cranioectodermal dysplasia 3	614099	AR
62	*IFT80*	611177	Short-rib thoracic dysplasia 2 with or without polydactyly	611263	AR
63	*IL1RN*	147679	Interleukin 1 receptor antagonist deficiency	612852	AR
63	*IL1RN*	147679	{Gastric cancer risk after H. pylori infection}	137215	AD

63	*IL1RN*	147679	{Microvascular complications of diabetes 4}	612628	-
64	*INPPL1*	600829	Opsismodysplasia	258480	AR
65	*LBR*	600024	Greenberg skeletal dysplasia	215140	AR
65	*LBR*	600024	Pelger-Huet anomaly	169400	AD
65	*LBR*	600024	Reynolds syndrome	613471	AD
66	*LEPRE1*	610339	Osteogenesis imperfecta, type V2I	610915	AR
67	*LIFR*	151443	Stuve-Wiedemann syndrome/Schwartz-Jampel type 2 syndrome	601559	AR
68	*LONP1*	605490	CODAS syndrome	600373	AR
69	*LRP5*	603506	Hyperostosis, endosteal	144750	AD
69	*LRP5*	603506	van Buchem disease, type 2	607636	AD
69	*LRP5*	603506	[Bone mineral density variability 1]	601884	AD
69	*LRP5*	603506	Osteopetrosis, autosomal dominant 1	607634	AD
69	*LRP5*	603506	{Osteoporosis}	166710	AD
69	*LRP5*	603506	Osteoporosis-pseudoglioma syndrome	259770	AR
69	*LRP5*	603506	Osteosclerosis	144750	AD
69	*LRP5*	603506	Exudative vitreoretinopathy 4	601813	AR, AD
70	*MAN2B1*	609458	Mannosidosis, alpha-, types I and 2	248500	AR
71	*MANBA*	609489	Mannosidosis, beta	248510	AR
72	*MATN3*	602109	{Osteoarthritis susceptibility 2}	140600	AD
72	*MATN3*	602109	Spondyloepimetaphyseal dysplasia	608728	AR
72	*MATN3*	602109	Epiphyseal dysplasia, multiple, 5	607078	AD
73	*MCOLN1*	605248	Mucolipidosis IV	252650	AR
74	*MMP13*	600108	Metaphyseal anadysplasia 1	602111	AD
74	*MMP13*	600108	Metaphyseal dysplasia, Spahr type	250400	AR
74	*MMP13*	600108	Spondyloepimetaphyseal dysplasia, Missouri type	602111	AD
75	*MMP9*	120361	Metaphyseal anadysplasia 2	613073	-
76	*NAGLU*	609701	Mucopolysaccharidosis type 2IB (Sanfilippo B)	252920	AR
76	*NAGLU*	609701	Charcot-Marie-Tooth disease, axonal, type 2V	616491	AD
77	*NEK1*	604588	Short-rib thoracic dysplasia 6 with or without polydactyly	263520	DR (Digenic recessive), AR
78	*NEU1*	608272	Sialidosis, type I	256550	AR
78	*NEU1*	608272	Sialidosis, type 2	256550	AR
79	*NME1*	156490	Neuroblastoma	256700	IC (Isolated cases), AD
80	*NKX3-2*	602183	Spondylo-megaepiphyseal-metaphyseal dysplasia	613330	AR
81	*NSDHL*	300275	CK syndrome	300831	XLR
81	*NSDHL*	300275	CHILD syndrome	308050	XLD
82	*PAPSS2*	603005	Brachyolmia 4 with mild epiphyseal and metaphyseal changes	612847	AR
83	*PEX1*	602136	Peroxisome biogenesis disorder 1B (NALD/IRD)	601539	-
83	*PEX1*	602136	Heimler syndrome 1	234580	AR
83	*PEX1*	602136	Peroxisome biogenesis disorder 1A (Zellweger)	214100	AR
84	*PEX5*	600414	Peroxisome biogenesis disorder 2B	202370	AR
84	*PEX5*	600414	Rhizomelic chondrodysplasia punctata, type 5	616716	AR
84	*PEX5*	600414	Peroxisome biogenesis disorder 2A (Zellweger)	214110	AR
85	*PEX6*	601498	Peroxisome biogenesis disorder 4B	614863	-
85	*PEX6*	601498	Heimler syndrome 2	616617	AR
85	*PEX6*	601498	Peroxisome biogenesis disorder 4A (Zellweger)	614862	-
86	*PEX7*	601757	Peroxisome biogenesis disorder 9B	614879	-
86	*PEX7*	601757	Rhizomelic chondrodysplasia punctata, type 1	215100	AR
87	*PHEX*	300550	Hypophosphatemic rickets, X-linked dominant	307800	XLD
88	*PHOSPHO1*	162466 (NCBI)	HAD Asp-based non-protein phosphatases	162466 (NCBI)	-
89	*PLOD2*	601865	Bruck syndrome 2	609220	AR
90	*PLS3*	300131	Bone mineral density QTL18, osteoporosis	300910	XLD
91	*PPIB*	123841	Osteogenesis imperfecta, type IX	259440	AR
92	*PTH1R*	168468	Eiken syndrome	600002	AR
92	*PTH1R*	168468	Metaphyseal chondrodysplasia, Murk Jansen type	156400	AD
92	*PTH1R*	168468	Failure of tooth eruption, primary	125350	AD
92	*PTH1R*	168468	Chondrodysplasia, Blomstrand type	215045	AR

93	*RAB33B*	605950	Smith-McCort dysplasia 2	615222	AR
94	*RUNX2*	600211	Cleidocranial dysplasia	119600	AD
94	*RUNX2*	600211	Cleidocranial dysplasia, forme fruste, with brachydactyly	119600	AD
94	*RUNX2*	600211	Cleidocranial dysplasia, forme fruste, dental anomalies only	119600	AD
94	*RUNX2*	600211	Metaphyseal dysplasia with maxillary hypoplasia with or without brachydactyly	156510	AD
95	*SBDS*	607444	Shwachman-Diamond syndrome	260400	AR
96	*SERPINF1*	172860	Osteogenesis imperfecta, type VI	613982	-
97	*SERPINH1*	600943	Osteogenesis imperfecta, type X	613848	AR
97	*SERPINH1*	600943	Preterm premature rupture of the membranes, susceptibility to	610504	-
98	*SGSH*	605270	Mucopolysaccharidosis type 2IA (Sanfilippo A)	252900	AR
99	*SLC17A5*	604322	Sialic acid storage disorder, infantile	269920	AR
99	*SLC17A5*	604322	Salla disease	604369	AR
100	*SLC26A2*	606718	De la Chapelle dysplasia	256050	AR
100	*SLC26A2*	606718	Achondrogenesis Ib	600972	AR
100	*SLC26A2*	606718	Atelosteogenesis 2	256050	AR
100	*SLC26A2*	606718	Diastrophic dysplasia	222600	AR
100	*SLC26A2*	606718	Diastrophic dysplasia, broad bone-platyspondylic variant	222600	AR
100	*SLC26A2*	606718	Epiphyseal dysplasia, multiple, 4	226900	AR
101	*SLC2A2*	138160	Diabetes mellitus, noninsulin-dependent	125853	AD
101	*SLC2A2*	138160	Fanconi-Bickel syndrome	227810	AR
102	*SLC34A1*	182309	Fanconi renotubular syndrome 2	613388	AR
102	*SLC34A1*	182309	Hypercalcemia, infantile, 2	616963	AR
102	*SLC34A1*	182309	Nephrolithiasis/osteoporosis, hypophosphatemic, 1	612286	AD
103	*SLC34A3*	609826	Hypophosphatemic rickets with hypercalciuria	241530	AR
104	*SLC35D1*	610804	Schneckenbecken dysplasia	269250	AR
105	*SLC4A1*	109270	Cryohydrocytosis	185020	AD
105	*SLC4A1*	109270	Ovalocytosis, SA type	166900	AD
105	*SLC4A1*	109270	Renal tubular acidosis, distal, AD	179800	AD
105	*SLC4A1*	109270	Renal tubular acidosis, distal, AR	611590	AR
105	*SLC4A1*	109270	Spherocytosis, type 4	612653	AD
106	*SLC4A4*	603345	Renal tubular acidosis, proximal, with ocular abnormalities	604278	AR
107	*SMARCAL1*	606622	Schimke immunoosseous dysplasia	242900	AR
108	*SOX9*	608160	Campomelic dysplasia	114290	AD
108	*SOX9*	608160	Campomelic dysplasia with autosomal sex reversal	114290	AD
108	*SOX9*	608160	Acampomelic campomelic dysplasia	114290	AD
109	*SP7*	606633	Osteogenesis imperfecta, type X2	613849	AR
110	*SPARC*	182120	Osteogenesis imperfecta, type XV2	616507	AR
111	*SPP1*	166490	Secreted phosphoprotein 1	-	-
112	*SUMF1*	607939	Multiple sulfatase deficiency	272200	AR
113	*TCTN3*	613847	Orofaciodigital syndrome IV	258860	AR
113	*TCTN3*	613847	Joubert syndrome 18	614815	AR
114	*TMEM38B*	611236	Osteogenesis imperfecta, type XIV	615066	-
115	*TRIP11*	604505	Achondrogenesis, type IA	200600	AR
116	*TRPV4*	605427	Brachyolmia type 3	113500	AD
116	*TRPV4*	605427	Digital arthropathy-brachydactyly, familial	606835	AD
116	*TRPV4*	605427	Metatropic dysplasia	156530	AD
116	*TRPV4*	605427	Hereditary motor and sensory neuropathy, type 2c	606071	AD
116	*TRPV4*	605427	Parastremmatic dwarfism	168400	AD
116	*TRPV4*	605427	Scapuloperoneal spinal muscular atrophy	181405	AD
116	*TRPV4*	605427	Spinal muscular atrophy, distal, congenital nonprogressive	600175	AD
116	*TRPV4*	605427	Spondylometaphyseal dysplasia, Kozlowski type	184252	AD
116	*TRPV4*	605427	SED (Spondyloepimetaphyseal Dysplasia), Maroteaux type	184095	AD
117	*TTC21B*	612014	Short-rib thoracic dysplasia 4 with or without	613819	AR

			polydactyly		
117	*TTC21B*	612014	Nephronophthisis 12	613820	AR, AD
118	*VDR*	601769	Rickets, vitamin D-resistant, type 2A	277440	AR
118	*VDR*	601769	Osteoporosis, involutional	166710	AD
119	*WDR19*	608151	Short-rib thoracic dysplasia 5 with or without polydactyly	614376	AR
119	*WDR19*	608151	Cranioectodermal dysplasia 4	614378	AR
119	*WDR19*	608151	Senior-Loken syndrome 8	616307	AR
119	*WDR19*	608151	Nephronophthisis 13	614377	AR
120	*WDR34*	613363	Short-rib thoracic dysplasia 11 with or without polydactyly	615633	AR
121	*WDR35*	613602	Short-rib thoracic dysplasia 7 with or without polydactyly	614091	AR
121	*WDR35*	613602	Cranioectodermal dysplasia 2	613610	AR
122	*WDR60*	615462	Short-rib thoracic dysplasia 8 with or without polydactyly	615503	AR
123	*WNT1*	164820	{Osteoporosis, early-onset, susceptibility to, autosomal dominant}	615221	-
123	*WNT1*	164820	Osteogenesis imperfecta, type XV	615220	AR
124	*RMRP*	157660	Cartilage-hair hypoplasia	250250	AR
124	*RMRP*	157660	Metaphyseal dysplasia without hypotrichosis	250460	AR
124	*RMRP*	157660	Anauxetic dysplasia	607095	AR
125	*FBN2*	612570	Contractural arachnodactyly, congenital	121050	AD
125	*FBN2*	612570	Macular degeneration, early-onset	616118	AD
126	*MYH3*	160720	Arthrogryposis, distal, type 2A	193700	AD
126	*MYH3*	160720	Arthrogryposis, distal, type 2B	601680	AD
126	*MYH3*	160720	Arthrogryposis, distal, type 8	178110	AD
127	*ROR2*	602337	Brachydactyly, type B1	113000	AD
127	*ROR2*	602337	Robinow syndrome, autosomal recessive	268310	AR
128	*BTK*	300300	Agammaglobulinemia and isolated hormone deficiency	307200	XLR
128	*BTK*	300300	Agammaglobulinemia, X-linked 1	300755	XLR
129	*GDF6*	601147	Klippel-Feil syndrome 1, autosomal dominant	118100	AD
129	*GDF6*	601147	Leber congenital amaurosis 17	615360	AR
129	*GDF6*	601147	Microphthalmia with coloboma 6, digenic	613703	-
129	*GDF6*	601147	Microphthalmia, isolated 4	613094	-
130	*GNAS*	139320	ACTH-independent macronodular adrenal hyperplasia	219080	IC (Isolated cases)
130	*GNAS*	139320	Osseous heteroplasia, progressive	166350	AD
130	*GNAS*	139320	Acromegaly, somatic	102200	-
130	*GNAS*	139320	Pseudohypoparathyroidism Ia	103580	AD
130	*GNAS*	139320	Pseudohypoparathyroidism Ib	603233	AD
130	*GNAS*	139320	Pseudohypoparathyroidism Ic	612462	AD
130	*GNAS*	139320	McCune-Albright syndrome, somatic, mosaic	174800	-
130	*GNAS*	139320	Pseudopseudohypoparathyroidism	612463	AD
131	*MSX2*	123101	Parietal foramina with cleidocranial dysplasia	168550	AD
131	*MSX2*	123101	Craniosynostosis 2	604757	AD
131	*MSX2*	123101	Parietal foramina 1	168500	AD
132	*TRIM37*	605073	Mulibrey nanism	253250	AR
133	*TCIRG1*	604592	Osteopetrosis, autosomal recessive 1	259700	AR
134	*GLI3*	165240	{Hypothalamic hamartomas, somatic}	241800	-
134	*GLI3*	165240	Greig cephalopolysyndactyly syndrome	175700	AD
134	*GLI3*	165240	Polydactyly, postaxial, types A1 and B	174200	AD
134	*GLI3*	165240	Polydactyly, preaxial, type IV	174700	AD
134	*GLI3*	165240	Pallister-Hall syndrome	146510	AD
135	*FGFR1*	136350	Hypogonadotropic hypogonadism 2 with or without anosmia	147950	AD
135	*FGFR1*	136350	Osteoglophonic dysplasia	166250	AD
135	*FGFR1*	136350	Jackson-Weiss syndrome	123150	AD
135	*FGFR1*	136350	Pfeiffer syndrome	101600	AD
135	*FGFR1*	136350	Hartsfield syndrome	615465	AD
135	*FGFR1*	136350	Trigonocephaly 1	190440	AD
135	*FGFR1*	136350	Encephalocraniocutaneous lipomatosis	613001	Smo (Somatic

					mosaicism)
136	*FGFR2*	176943	Craniofacial-skeletal-dermatologic dysplasia	101600	AD
136	*FGFR2*	176943	Craniosynostosis, nonspecific	-	-
136	*FGFR2*	176943	Gastric cancer, somatic	613659	-
136	*FGFR2*	176943	Antley-Bixler syndrome without genital anomalies or disordered steroidogenesis	207410	AR
136	*FGFR2*	176943	Apert syndrome	101200	AD
136	*FGFR2*	176943	Beare-Stevenson cutis gyrata syndrome	123790	AD
136	*FGFR2*	176943	Jackson-Weiss syndrome	123150	AD
136	*FGFR2*	176943	Pfeiffer syndrome	101600	AD
136	*FGFR2*	176943	Saethre-Chotzen syndrome	101400	AD
136	*FGFR2*	176943	Scaphocephaly and Axenfeld-Rieger anomaly	-	-
136	*FGFR2*	176943	Scaphocephaly, maxillary retrusion, and mental retardation	609579	-
136	*FGFR2*	176943	Crouzon syndrome	123500	AD
136	*FGFR2*	176943	Bent bone dysplasia syndrome	614592	AD
136	*FGFR2*	176943	LADD syndrome	149730	AD
137	*HOXD13*	142989	Brachydactyly, type D	113200	AD
137	*HOXD13*	142989	Brachydactyly, type E	113300	AD
137	*HOXD13*	142989	?Brachydactyly-syndactyly syndrome	610713	-
137	*HOXD13*	142989	Syndactyly, type V	186300	AD
137	*HOXD13*	142989	Synpolydactyly 1	186000	AD
138	*ROBO3*	608630	Gaze palsy, horizontal, with progressive scoliosis	607313	AR
139	*DLL3*	602768	Spondylocostal dysostosis 1, autosomal recessive	277300	AR
140	*TRAPPC2*	300202	Spondyloepiphyseal dysplasia tarda	313400	XLR
141	*ACVR1*	102576	Fibrodysplasia ossificans progressiva	135100	AD
142	*ERCC6*	609413	{Macular degeneration, age-related, susceptibility to 5}	613761	-
142	*ERCC6*	609413	Premature ovarian failure 11	616946	AD
142	*ERCC6*	609413	{Lung cancer, susceptibility to}	211980	AR
142	*ERCC6*	609413	Cockayne syndrome, type B	133540	AR
142	*ERCC6*	609413	De Sanctis-Cacchione syndrome	278800	AR
142	*ERCC6*	609413	UV-sensitive syndrome 1	600630	AR
142	*ERCC6*	609413	Cerebrooculofacioskeletal syndrome 1	214150	AR
143	*HEXB*	606873	Sandhoff disease, infantile, juvenile, and adult forms	268800	AR
144	*HEXA*	606869	Tay-Sachs disease	272800	AR
144	*HEXA*	606869	[Hex A pseudodeficiency]	272800	AR
144	*HEXA*	606869	GM2-gangliosidosis, several forms	272800	AR
145	*HYAL1*	607071	Mucopolysaccharidosis type IX	601492	AR
146	*GM2A*	613109	GM2-gangliosidosis, AB variant	272750	AR
147	*AP2S1*	602242	Hypocalciuric hypercalcemia, familial, type 2I	600740	AD
148	*CASR*	601199	Hypocalciuric hypercalcemia, type I	145980	AD
148	*CASR*	601199	Hypercalciuric hypercalcemia	-	-
148	*CASR*	601199	Hyperparathyroidism, neonatal	239200	AR
148	*CASR*	601199	Hypocalcemia, autosomal dominant	601198	AD
148	*CASR*	601199	Hypocalcemia, autosomal dominant, with Bartter syndrome	601198	AD
148	*CASR*	601199	{Epilepsy idiopathic generalized, susceptibility to, 8}	612899	-
148	*CASR*	601199	{Calcium, serum level of}	-	-
149	*GNA11*	139313	Hypocalcemia, autosomal dominant 2	615361	AD
149	*GNA11*	139313	Hypocalciuric hypercalcemia, type 2	145981	AD
150	*SLC9A3R1*	604990	Nephrolithiasis/osteoporosis, hypophosphatemic, 2	612287	AD
151	*CLCN7*	602727	Osteopetrosis, autosomal recessive 4	611490	АР
151	*CLCN7*	602727	Osteopetrosis, autosomal dominant 2	166600	АД
152	*GJA1*	121014	Atrioventricular septal defect 3	600309	АД
152	*GJA1*	121014	Craniometaphyseal dysplasia, autosomal recessive	218400	АР
152	*GJA1*	121014	Palmoplantar keratoderma with congenital alopecia	104100	АД
152	*GJA1*	121014	Hypoplastic left heart syndrome 1	241550	АР
152	*GJA1*	121014	Oculodentodigital dysplasia	164200	АД
152	*GJA1*	121014	Oculodentodigital dysplasia	257850	АР
152	*GJA1*	121014	Syndactyly, type 2I	186100	АД

152	*GJA1*	121014	Erythrokeratodermia variabilis et progressiva 3	617525	АД
153	*HPGD*	601688	Digital clubbing, isolated congenital	119900	АР
153	*HPGD*	601688	Hypertrophic osteoarthropathy, primary, autosomal recessive 1	259100	АР
153	*HPGD*	601688	Cranioosteoarthropathy	259100	АР
154	*MTAP*	156540	Diaphyseal medullary stenosis with malignant fibrous histiocytoma	112250	АД
155	*OSTM1*	607649	Osteopetrosis, autosomal recessive 5	259720	АР
156	*PLEKHM1*	611466	Osteopetrosis, autosomal recessive 6	611497	АР
157	*PTDSS1*	612792	Lenz-Majewski hyperostotic dwarfism	151050	АД
158	*SLCO2A1*	601460	Hypertrophic osteoarthropathy, primary, autosomal recessive 2	614441	АР
159	*SNX10*	614780	Osteopetrosis, autosomal recessive 8	615085	АР
160	*SOST*	605740	Van Buchem disease	239100	АР
160	*SOST*	605740	Craniodiaphyseal dysplasia, autosomal dominant	122860	АД
160	*SOST*	605740	Sclerosteosis 1	269500	АР
161	*TBXAS1*	274180	?Thromboxane synthase deficiency	614158	АД
161	*TBXAS1*	274180	Ghosal hematodiaphyseal syndrome	231095	АР
162	*TGFB1*	190180	Camurati-Engelmann disease	131300	АД
163	*TNFRSF11A*	603499	{Paget disease of bone 2, early-onset}	602080	АД
163	*TNFRSF11A*	603499	Osteolysis, familial expansile	174810	АД
163	*TNFRSF11A*	603499	Osteopetrosis, autosomal recessive 7	612301	АР
164	*TNFRSF11B*	602643	Paget disease of bone 5, juvenile-onset	239000	АР
165	*TNFSF11*	602642	Osteopetrosis, autosomal recessive 2	259710	AR
166	*TYROBP*	604142	Nasu-Hakola disease	221770	AR
